# Neo4j graph dataset of cycling paths in Slovenia

**DOI:** 10.1016/j.dib.2023.109251

**Published:** 2023-05-27

**Authors:** Alen Rajšp, Iztok Fister

**Affiliations:** Faculty of Electrical Engineering and Computer Science, University of Maribor, Koroška cesta 46, Maribor SI-2000, Slovenia

**Keywords:** Data mining, Geographical data, Graph database, OpenStreetMap, Route generation, Sports training

## Abstract

Navigating through a real-world map can be represented in a bi-directed graph with a group of nodes representing the intersections and edges representing the roads between them. In cycling, we can plan training as a group of nodes and edges the athlete must cover. Optimizing routes using artificial intelligence is a well-studied phenomenon. Much work has been done on finding the quickest and shortest paths between two points. In cycling, the solution is not necessarily the shortest and quickest path. However, the optimum path is the one where a cyclist covers the suitable distance, ascent, and descent based on his/her training parameters. This paper presents a Neo4j graph-based dataset of cycling routes in Slovenia. It consists of 152,659 nodes representing individual road intersections and 410,922 edges representing the roads between them. The dataset allows the researchers to develop and optimize cycling training generation algorithms, where distance, ascent, descent, and road type are considered.

**Specifications Table**

**Table:** 

Subject	Computer Science
Specific subject area	Data Mining, Smart Sport Training
Type of data	Graph database dataset
How the data were acquired	Data for the construction of a graph database model were acquired through data fusion of OpenStreetMap [1] geographical database and EU DEM [2] Digital Surface Model. The Overpass API [3] was used for work with OpenStreetMap, and OpenElevation API [4] was used for querying the EU DEM model.
Data format	Raw: Neo4J database dump Raw: MongoDb database dump
Description of data collection	The processed data were limited to the area of Slovenia. The OpenStreetMap geographical data were taken from Geofabrik Slovenia map [5] created on the 23rd of September 2022, study [6] has shown that OpenStreetMap geographical data individual node locations are highly accurate and, on average, located a maximum of 6 m from their actual locations. The E40N20 region from EU DEM [2] elevation data was used because the whole territory of Slovenia was in the address. The elevations are sampled in 25-meter squares and are highly accurate. The statistical validation of the EU-DEM model [7] has shown that locations in Slovenia have a mean error of −0.40 m (Standard Deviation: 1.64 m, Root Mean Square Error: 1.69 m).
Data source location	Secondary data - EU-DEM Digital Surface model [2] Secondary data - OpenStreetMap [5]
Data accessibility	Repository name: Mendeley Data^1^ Data identification number: 10.17632/zkbfvsjr5f.2 Direct URL to data: https://doi.org/10.17632/zkbfvsjr5f.2 Alternative repository name: GitHub^2^ Repository identification id: **firefly-cpp/osm-graph** Direct URL to data: https://github.com/firefly-cpp/osm-graph
Related research article	A. Rajšp, M. Heričko, and I. Fister Jr., ”Preprocessing of roads in OpenStreetMap based geographic data on a property graph,” in Proceedings of the Central European Conference on Information and Intelligent Systems, 2921, pp. 193–199. Accessed: Apr. 17, 2023. [Online]. Available: https://www.proquest.com/docview/2604879635/

## Value of the Data

• This graph database dataset enables the generation of cycling routes and the development of new computational intelligence algorithms for cycling training route generation.
• Machine learning, data mining and Artificial Intelligence researchers can use this dataset to develop path-finding algorithms and discover relations between the connectedness of the roads suitable for cycling.
• This dataset can be used as a performance benchmark for developing path-finding algorithms that have to generate multi-parameter optimized paths.

## 1 Objective

This research paper provides the generated data prepared by following the method proposed in [8]. The generated graph database demonstrates the method's viability on larger scales (e.g., generating whole-country graph database maps). The article and the corresponding data enable further use of the graph database dataset without requiring the implementation of the method.

## 2 Data Description

The presented dataset provides two files, Neo4J database dump (file **slovenia-graph-neo4j.dump**) and MongoDB database dump (**pathways.json**). The repository also contains two folders, one for instructions on creating your own cycling paths dataset from scratch and another for providing examples on using the provided dataset for finding a sample route between two points.

## 3 Neo4J Database

Neo4j database dump contains a graph database with all the intersections and roads suitable for cycling in Slovenia. The data is composed of edges and relationships between them. The edges represent individual road/path intersections, and relationships represent the roads/paths between them. The graph database contains 152,659 intersections and 410,922 paths between them.

Each intersection (Table 1) saved in the graph contains latitude, longitude, node id (which is an OpenStreetMap identificator of the intersection), and way ids represent the OpenStreetMap ways the intersection belongs to.

**Table 1:** Neo4j node (intersection).

Intersection		
Attribute	Description	Example value
latitude	Geographical latitude	46.1036427
longitude	Geographical longitude	15.6247595
node id	OpenStreetMap node id	1017223619
way_ids	OpenStreetMap way ids the node is part of	[92405204, 844360092]

The intersections are connected with paths. Each path contains ascent, descent, distance, and type values (Table 2). Because traveling from intersection A to intersection B is not the same as traveling from B to A, the relationships are directed due to the ascent and descent between them.

**Table 2:** Neo4j relationship (path).

Path		
Attribute	Description	Example value
ascent	Ascent in meters	71
descent	Descent in meters	7
Distance	Distance in meters	328.22
Type	OpenStreetMap road type	Track
*Intersection-AB*	*Relationship from - to node ids*	4893616681–4893616518

## 4 MongoDB Database

**Fig. 1:**
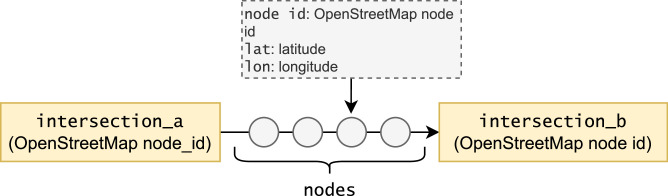
MongoDB data.

The data for nodes of each way is saved inside a MongoDB database dump (pathways.json), as seen in Fig. 1. The collection *pathways* contain a list of all relationships. Each object contains the route's starting node id (intersection a) and the ending node id (intersection b). The object also contains a list of all relationship nodes and their latitudes and longitudes.

## 5 Experimental Design, Materials and Methods

**Fig. 2:**
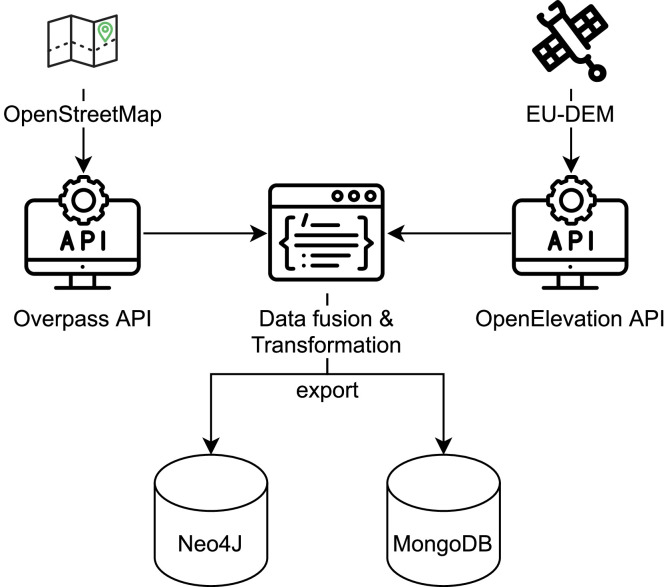
Data fusion graph generation diagram.

The solution for generating graph databases from geospatial data was first proposed in [8]. The original method (1) identified all the intersections, (2) identified ways (routes) between them, and during the second step, the distances, as well as elevations, were calculated and saved to the Neo4J database. For each way, nodes with latitude and longitude pairs were identified and saved to the MongoDB database to enable data visualization. The architecture of the solution is presented in Fig 2.

Generating your own Neo4j based cycling paths property graph

The Mendeley Data repository presented in this article also contains a simple four-step procedure for generating the Neo4J cycling property graph, together with the documentation of use. To successfully generate the property graph, the user must first prepare the necessary environment by installing and self-hosting the OpenElevation API, Overpass API, and two Redis database instances as well as one Neo4j database instance. Once done, he needs to download the project and launch the following four Python scripts (located in the *creating_Your_own_property_graph/example_workflow* folder) in sequential order:

• A_intersection_db_parser.py – The script executes the prepared Overpass API queries in the given area and identifies the intersections, which are saved into a Redis database
• B_intersections_to_neo4j_graph.py – The script imports Redis saved intersections into a Neo4j database (Table 1) and queries individually identified intersections for valid paths.
• C_connections_to_graph.py – The script executes the Overpass API queries to find links between nodes and establish relationships objects (Table 2).
• D_merge.py – This step is optional. It serves to merge paths and intersections where less than two relationships originate from an intersection.

## 6 Sample Use

Two cases of using the database are presented on the Git-Hub repository (https://github.com/firefly-cpp/osm-graph). The first case demonstrates saving the database into a binary file using pickle. Moreover, the second example showcases importing the generated graph into a Python library igraph network analysis package [9] object is demonstrated, where a sample shortest route is generated between two intersections.

## Ethics Statements

This work did not involve human subjects, animal experiments, and data collected from social media platforms.

## CRediT authorship contribution statement

**Alen Rajšp:** Conceptualization, Methodology, Software, Writing – review & editing. **Iztok Fister:** Conceptualization, Project administration, Supervision.

## Declaration of Competing Interest

The authors declare that they have no known competing financial interests or personal relationships that could have appeared to influence the work reported in this paper.

## Footnotes

1 Contains the Neo4j graph dataset (version 23rd September 2022).

2 Contains the Neo4j graph dataset (version 23rd September 2022) and also up to date code examples.
